# Microbial mimics supersize the pathogenic self-response

**DOI:** 10.1172/JCI184046

**Published:** 2024-09-17

**Authors:** Jesusa Capera, Michael L. Dustin

**Affiliations:** Kennedy Institute of Rheumatology, University of Oxford, Oxford, United Kingdom.

## Abstract

Microbial mimicry, the process in which a microbial antigen elicits an immune response and breaks tolerance to a structurally related self-antigen, has long been proposed as a mechanism in autoimmunity. In this issue of the *JCI*, Dolton et al. extend this paradigm by demonstrating that a naturally processed peptide from *Klebsiella oxytoca* acts as a superagonist for autoreactive T cells in type 1 diabetes (T1D). Reframing microbial mimics as superagonists that are thousands of times better at binding disease-associated autoreactive T cell receptors than self-peptides serves to narrow the search space for relevant sequences in the vast microbial proteome. Moreover, the identified superagonists have implications for the intervention and personalized monitoring of T1D that may carry over to other autoimmune diseases with microbial mimicry.

## Peptides that bind a diabetes-associated TCR

Type 1 diabetes (T1D) is an autoimmune disease characterized by the destruction of the insulin-producing β cells in the pancreatic islets of Langerhans by mechanisms including CD8^+^ T cells specific for preproinsulin (PPI), leading to insulin deficiency and hyperglycemia at an early age (1). A combination of environmental and genetic factors is involved. High-risk genes include certain human leucocyte antigen (HLA) alleles and environmental factors, including dietary intake, viral exposure, and the microbiome profile. Pancreatic infiltrates in diabetes include both CD4^+^ and CD8^+^ T cells, the last being the main population, and autoantibodies are also produced. Key targeted self-antigens expressed by β cells include insulin, glutamic acid decarboxylase (GAD), islet antigen-2 (IA-2), and zinc transporter 8 (ZnT8) (2). Molecular mimicry is one of a number of mechanisms that have been proposed to break tolerance to these self-antigens (3).

In this issue of the *JCI*, Dolton et al. (4) focused on the previously characterized 4C6 T cell receptor (TCR), which recognizes diabetes-associated HLA A*24:02 in complex with the peptide sequence LWMRLLPLL, a self-peptide from PPI. The 4C6 TCR targets and kills HLA A*24:02–expressing human β cells and is associated with an earlier onset of the disease (5). Using a combinatorial peptide library, Dolton, Bulek, and colleagues characterized the peptide recognition landscape of the 4C6 TCR. This approach enabled them to predict and identify peptide ligands capable of binding to the 4C6 TCR. Notably, peptides forming complexes with HLA A*24:02 that bound the 4C6 TCR more potently than the natural insulin peptide were deemed superagonists (4).

The authors crystallized the 4C6 TCR in complex with the HLA A*24:02 superagonistic peptide and used these crystals to seed complexes with the PPI self-peptide. This process allowed them to generate the first atomic-resolution structure of a self-peptide–HLA A*24:02 complex binding to its TCR. This approach, analogous to the practice of soaking small molecule ligands into existing crystals, may enable analysis of other difficult-to-crystalize autoantigens. Despite the self-peptide and the microbial superagonistic HLA A*24:02 peptide showing different affinities for the 4C6 TCR, the structural data revealed both peptides formed complexes and bound the 4C6 TCR in identical orientations (4).

Prior crystallographic results with MHC class II–presented self-peptides suggested atypical binding orientations (6). The orientation of the TCR with respect to the peptide may be more strongly constrained in MHC class I by the role of CD8. A caveat is that it remains possible that the method of in-crystal peptide exchange may not allow changes in conformation that might otherwise happen due to dominance of other crystal contacts, so other approaches to obtaining structural information about the self-peptide complexes might possibly yield different structures. By combining the structural data with the combinatorial peptide library, Dolton et al. (4) identified the consensus peptide sequence recognized by the 4C6 TCR: any 9-mer peptide with the motif X-H/K/L/M/N/W/Y-X-P/R-L-X-X-X-A/F/I/L/M/V/W. This finding indicates that 4C6 T cells have a high potential for cross-reactivity with numerous peptide sequences, but the selection for predicted superagonists allowed the authors to focus on selected peptides in the context of microbial proteomes that potentially contain thousands of proteins.

**Figure 1:**
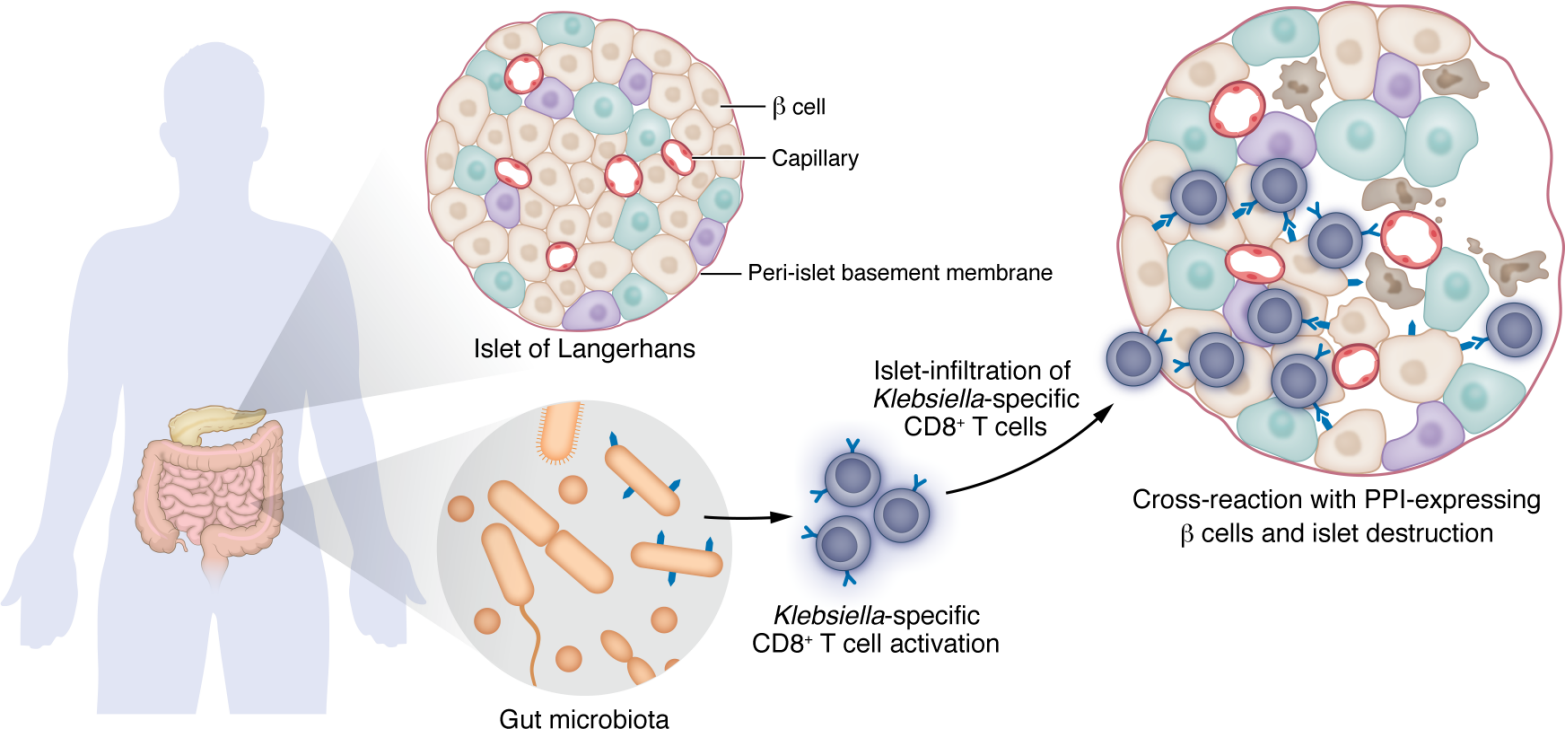
A working model for molecular mimicry driving T1D through microbiota-derived peptide–driven T cell responses. A *Klebsiella oxytoca* peptide produced in the gut activates CD8^+^ T cells, which then differentiate and travel to the pancreas. In pancreatic islets, *Klebsiella*-specific CD8^+^ T cells cross-react with the HLA A*24:02–peptide complex on PPI-expressing β cells. Cytotoxic T cell activity results in islet destruction and ultimately manifests as diabetes.

Indeed, pathogen-derived peptides were identified from fungal and bacterial species, which were thousands of times more potent at activating the 4C6 TCR than the PPI self-peptide and matched the consensus sequence identified by the authors. From all these potentially pathogenic peptides, the strongest activating bacterial ligand tested was the SLPRLFPLL peptide derived from *Klebsiella oxytoca* (30,000 times more potent than the natural PPI peptide), leading to a model for molecular mimicry as a driver for CD8^+^ T cell response in T1D (Figure 1). Dolton et al. (4) show that PPI-specific CD8^+^ T cells from different patients with diabetes can recognize the *Klebsiella* peptide–HLA A*24 complex. On the other side, *Klebsiella*-specific CD8^+^ T cells from healthy HLA A*24^+^ donors can recognize and kill surrogate pancreatic cells by recognition of the PPI peptide. In fact, Dolton, Bulek, and authors showed that cross-reactive *Klebsiella*-specific TCRs from healthy donors possessed sequences with similarities to the T1D-associated 4C6 TCR (4).

## Diabetes as a possible pathogen-induced autoimmune disease

Molecular mimicry has been proposed as the mechanism triggering the disruption of self-tolerance that precedes the onset of various autoimmune diseases. This hypothesis suggests that foreign antigens resembling epitopes on native human proteins can activate cross-reactive lymphocytes, which then mistakenly attack the body’s own tissues expressing these mimicked self-antigens. The best example to date for a causal relationship between a pathogen and an autoimmune disease is in multiple sclerosis (MS). In 1995, Wucherpfennig and Strominger (7) identified Epstein-Barr virus (EBV) epitopes, among other viral and bacterial sources, that were cross-reactive with disease-associated CD4^+^ T cell clones from patients with MS. This work identified a set of candidates, but it then took over 25 years to develop a compelling case for a causal relationship between EBV and MS (8, 9). Analogous mechanisms involving various autoimmune diseases and different viral or bacterial antigens have also been proposed (3). In the case of T1D, some viruses and commensal gut bacteria express proteins that can generate effective T cell epitopes for a diabetogenic TCR recognizing self-antigens such as GAD56, IA-2, and ZnT8 (3).

Yet, a pivotal question persists in challenging the molecular mimicry hypothesis for autoimmunity: If cross-reactivity initiates the autoinflammatory process, why do only a minority of individuals infected by the same pathogen, with the relevant MHC alleles, microbial exposure, and even detectable autoreactive T cells, never develop disease? Simply put, molecular mimicry to expand T cells with self-reactive TCR may be necessary, but not sufficient, for pathological autoimmunity. Furthermore, the sequence requirements to initiate pathogenesis may be more extensive than the consensus peptide identified in Dolton et al. (4). A complex cascade of genetic and environmental conditions is likely to be required before and after the expansion of cross-autoreactive T cells for the onset of the clinical autoimmune disease, including infiltration of the islets of Langerhans, a location that normally has few T cells (Figure 1). Moreover, even if such autoreactive T cell clones are expanded, numerous peripheral tolerance mechanisms may swiftly intervene to prevent them from becoming pathogenic (10).

## The evolutionary trade-off of molecular mimicry

Any mechanism leading to an autoimmune response is pathological and potentially life-threatening for the individual. However, cross-reactivity is an intrinsic property of adaptive immune systems that need to protect us against a never-ending plethora of co-evolving pathogens. Population-wise, the degeneracy of T cell recognition that underlies molecular mimicry substantially broadens the antigen repertoire that our immune system can recognize and attack, thereby providing a survival benefit that outweighs the potential risks associated with a rare collateral autoreactive response, which requires additional promoting factors to develop. On the other hand, molecular mimicry presents both advantages and risks for the pathogen. By disguising as “self,” the pathogen can evade the immune system, gaining a clear survival advantage. However, this mimicry also poses risks. If an autoimmune response is triggered due to the cross-reaction event, the resulting inflammatory process can lead to the clearance of the pathogen itself, in addition to the destruction of the host’s own targeted tissue. It has been noted that individuals with more resilient T cell response profiles are better able to resist viral pathogens, but display greater severity of autoimmunity (11).

Mimicry offers additional potential benefits for the host. Epitopes from commensal bacteria can cross-react with cancer neoantigens, providing a protective effect against the oncogenic process (12, 13). In fact, the relationship between the gut microbiota and cancer development is well documented, with tumor-mimicking antigens likely being one of the mechanisms by which commensal bacteria confer these benefits (14, 15). Furthermore, pathogenic microorganisms might also offer cancer protection through cross-reactivity with oncogenic antigens. For instance, *Helicobacter pylori* infection has been associated with a decreased risk of esophageal adenocarcinoma (16). However, further research is needed to determine whether this, or other infectious agents, can trigger a protective cross-reaction response against cancer. Therefore, molecular mimicry constitutes a crucial bidirectional mechanism that shapes the delicate balance between host and pathogen.

## Clinical relevance of a pathogen-derived origin in T1D

*Klebsiella oxytoca* is a commensal microorganism that confers colonization resistance and protection against other infectious agents (17). However, gut dysbiosis can lead to *Klebsiella* infections, potentially resulting in more severe conditions. The high cross-reactivity between a *Klebsiella oxytoca* peptide and a T1D-associated antigen presents a promising avenue for the treatment and prevention of this autoimmune disease by means of maintaining or restoring eubiosis. In fact, the use of prebiotics and probiotics in T1D is currently under intense study (18). Given the extensive list of possible microorganisms mimicking T1D antigens described by Dolton et al., many other commensal bacteria and pathogens besides *Klebsiella oxytoca* could be relevant. Therefore, a deeper understanding of the relationship between the gut microbiota and T1D will undoubtedly allow us to improve the disease prognosis. Furthermore, identifying the specific pathogen that triggers the autoimmune response is essential for developing vaccines that prevent the autoimmune reaction by targeting the mimicking agent. For example, vaccines against Coxsackievirus B are under study, as this enterovirus shares homology with the T1D-associated autoantigen GAD65 and it has been epidemiologically linked with the development of T1D (19).

Identification of T1D-associated pathogens also opens avenues for more personalized disease monitoring. By assessing individual risk profiles for T1D development alongside microbiota composition and previous pathogen exposures, proactive measures could be implemented to prevent or manage the disease effectively. For instance, monitoring antibodies against potential pathogens could serve as biomarkers to track disease progression or predict future outbreaks, even before clinical symptoms manifest. This would allow timely interventions and treatments that could substantially improve disease outcomes.

Another important finding from Dolton et al.’s research (4), beyond the identification of the consensus sequence for the 4C6 TCR–activating peptide that ultimately triggers β cell destruction, is the discovery of superagonists that are more likely to be recognized by immunodominant, pathogen-specific T cells. While this result may have specific applications in de-risking vaccines (20), the general observation that autoreactive T cells display attenuated recognition compared with pathogen-specific T cells (21) opens the space to improve prediction of potentially causal microbes. Therefore, the work by Dolton et al. (4) paves the way for a more personalized approach to prevent, diagnose, and treat T1D and other autoimmune diseases.

## Limitations of the study and next questions

The observation that *Klebsiella*-derived peptides can cross-react with PPI-specific CD8^+^ T cells, and vice versa, does not demonstrate that this interaction occurs in vivo or that it effectively triggers an autoreactive response. In fact, islet-reactive CD8^+^ T cells are found in the peripheral circulation at frequencies in healthy individuals similar to those with T1D (22), and *Klebsiella* is a common commensal bacterium present in many healthy individuals who will never develop T1D. The authors acknowledge that they chose the *Klebsiella* candidate as appearing to be the best of several options, and have not found any basis to reject it as a candidate, but the other candidates that were not taken forward should be kept in mind for future investigation of causal microbes. Further investigation is necessary to elucidate the factors in the host-pathogen interaction that may tip the balance and potentially lead to autoimmunity.

Additional epidemiological and clinical evidence is required to establish a causal relationship between microbes and T1D specifically, and non-MS autoimmunity generally. Nonetheless, with many large T1D studies ongoing (1), the predictions of Dolton et al. (4) can be tested with implications for disease prevention and prognosis. Dolton et al.’s conceptual and technical innovations may enable better predictions connecting microbes and autoantigens across other autoimmune processes.

## Version 1 09/17/2024

Electronic publication
